# Evaluation of 644 Percutaneous Endoscopic Gastrostomy Patients in a Single Center

**DOI:** 10.7759/cureus.38324

**Published:** 2023-04-30

**Authors:** Umut firat Turan, Mehmet Kağan Katar

**Affiliations:** 1 General Surgery, Atlas University, Istanbul, TUR

**Keywords:** peg, complications, risk factors, outcome, percutaneous endoscopic gastrostomy

## Abstract

Objective: Our study aimed to review and evaluate the indications, complications, complication-related risk factors, and mortality rates of percutaneous endoscopic gastrostomy (PEG) performed in a single university hospital.

Methods: We retrospectively examined hospital records of all 819 patients who underwent PEG between January 2010 and January 2019. Patients whose information was not available for various reasons, who had a history of gastrectomy, who were under 18 years old, and/or who had undergone PEG before, were excluded from the study.

Results: The mean age of the patients was 65.12 ± 15.42 years, and the majority of the patients (60.6%) were female. In the vast majority of patients, the PEG indication was due to neurological causes (71.5%), among which the majority was a stroke. The overall complication rate in our study was 11.2%. The most common was a peristomal infection in 37 (5.7%) patients. Patients who were not under any antibiotic treatment and/or had diabetes mellitus had a higher risk for peristomal infection. Dementia increased the risk of tube dislodgement. The use of clopidogrel, the simultaneous use of aspirin and clopidogrel, and hypertension were independent risk factors for bleeding complications. The one-year mortality risk was significantly higher in patients who underwent PEG due to neurological causes compared to those who underwent PEG due to malignancy or other reasons (*p* = 0.021, *p* = 0.038, respectively).

Conclusion: The PEG procedure is a safe and feasible technique due to its low complication and mortality rate in patients with swallowing disorders who need long-term nutritional support.

## Introduction

Percutaneous endoscopic gastrostomy (PEG), which was first described by Gauderer et al. in 1980, is the method used to provide enteral nutrition to patients who have a functional gastrointestinal tract and need long-term nutritional support due to swallowing disturbances caused by neurological impairment, dementia, trauma, or malignant disease [1,2]. Technically, there are three methods in clinical practice: the ‘pull’ technique, the ‘push’ (guidewire) technique, and the introducer (Russell) method [3,4]. Among these, the most widely used today is the ‘pull’ technique as defined by Gauderer et al. [1,2].

Insertion of the gastrostomy tube can be performed endoscopically, surgically, or radiologically. PEG has some advantages compared to surgical gastrostomies, such as a lower complication rate, faster implementation, and fewer required medical resources [5]. Although some studies have reported that there are minor complications in percutaneous radiological gastrostomy compared to PEG, neither method has a clear advantage over the other [6,7]. On the other hand, in a meta-analysis involving 11 randomized controlled studies, there was no difference between nasogastric tubes and PEG in terms of complications such as mortality and aspiration pneumonia. In contrast, PEG was more effective and safer due to the lower probability of intervention failure [8].

Although PEG has some advantages over other techniques, there are some life-threatening complications, such as bleeding, internal organ damage, necrotizing fasciitis, and aspiration pneumonia, as well as minor complications, including pneumoperitoneum, PEG site herniation, wound infection, tube dislodgement, and peristomal leakage [9,10]. In addition, one study reported a one-year mortality rate of 35.8%, while another showed a six-month mortality rate of 51.9% [11,12].

Our study aimed to review and evaluate the indications, complications, complication-related risk factors, and mortality rates of PEG performed in a single university hospital.

## Materials and methods

Patients and study design

**Figure 1:**
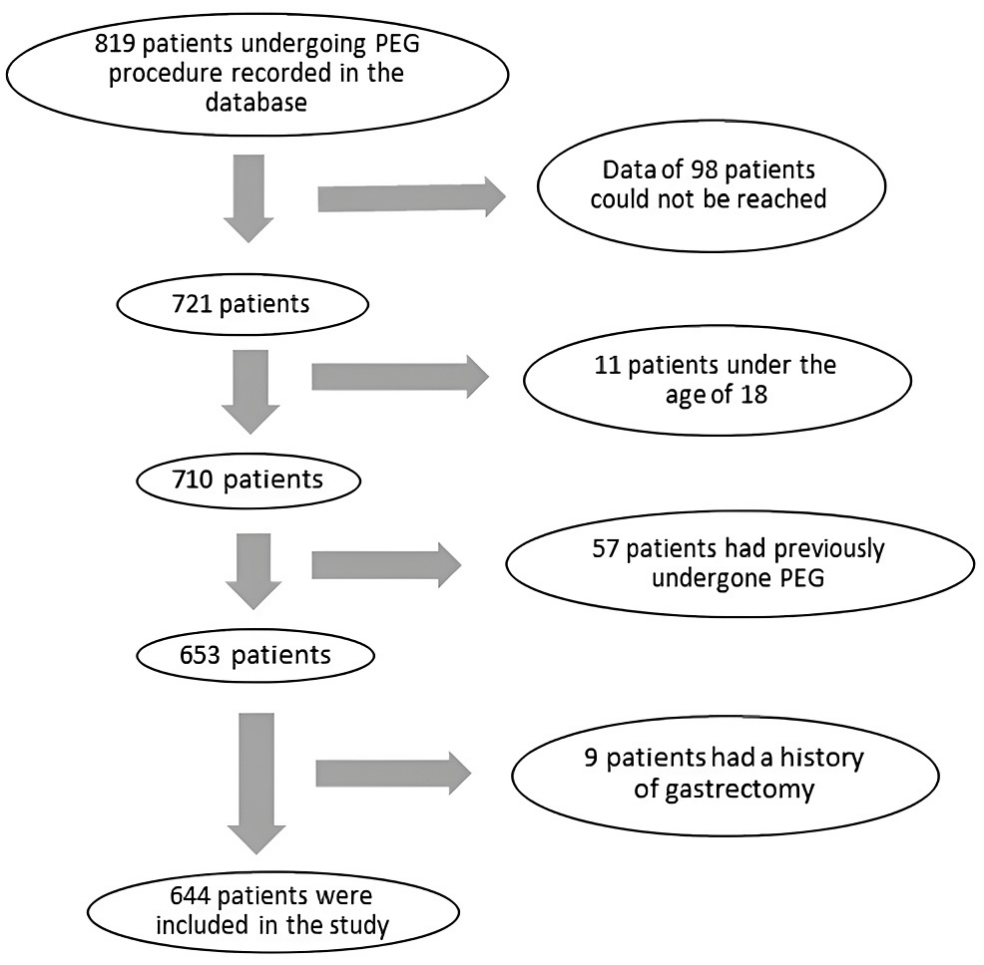
Study flow chart. PEG: percutaneous endoscopic gastrostomy.

This retrospectively designed study was performed according to the Declaration of Helsinki after the necessary ethics committee approval was received from the Clinical Research Ethics Committee of Yozgat Bozok University (IRB approval number: 2017-KAEK-189_2019.11.27_10). We examined the hospital records of all 819 patients who underwent PEG between January 2010 and January 2019. We also investigated developments during the first one-year follow-up of the patients after the PEG procedure via telephone conversations with their relatives. Patients whose information was not available for various reasons, who had a history of gastrectomy, who were under 18 years old, and/or who had undergone PEG before, were excluded from the study. The remaining 644 patients were included in the study after the exclusion criteria were applied. A flow chart of the study population is presented in Figure 1.

The data obtained from the patient’s medical records included demographic information, comorbidity conditions, PEG process indications, sedative drugs used during the PEG tube-insertion procedure, data on the administration of anticoagulant, antiplatelet, and antibiotic drugs, complications associated with the PEG procedure, and mortality information. PEG-related complications and mortality were recorded within one year after the PEG procedure.

PEG procedure

The PEG procedure was performed either in the endoscopy unit or at the patient’s bedside. Before the operation, patients and/or relatives were informed about the PEG procedure and its complications, and written consent was routinely received.

The oral intake of patients was confirmed by medical records, where the procedure was terminated six hours before the scheduled time. Routine antibiotic prophylaxis was not applied before the procedure; it was adjusted according to the physician’s preference. In patients who received prophylactic antibiotics, 1 g of cefazolin was given intravenously one hour before the procedure. In some patients who were considered to be at high risk for bleeding complications, anticoagulant and antiaggregant therapies were discontinued before the PEG procedure [13]. A sedation order of one mg/kg propofol (propofol-Lipuro 1% Brains, Istanbul, Turkey) or 0.05 mg/kg midazolam was administered, and prilocaine was used for local anaesthesia. All patients who were not intubated were given oxygen from the nasal cannula at 2 L/min during the procedure. Heart rate, blood pressure, and oxygen saturation for all patients were monitored.

The Ponsky-Gauderer (pull) technique was used for the PEG procedure in all patients [1]. In this technique, the PEG procedure begins with the transillumination of the endoscope in the abdomen to determine the region to open the PEG. The guidewire placed at the puncture site is taken out of the mouth with endoscopic biopsy forceps. The PEG tube is pulled out of the abdomen after it is connected to the guidewire. Finally, the endoscopic examination is repeated to confirm the placement of the tube.

Adverse events definitions

We defined a peristomal infection as erythema, tenderness, oedema, temperature increase, and/or pus discharge in the puncture site. We described bleeding as a haemorrhage requiring interventional procedures (such as hemoclip application or epinephrine injection), bleeding due to a mucosal tear, or bleeding with compression during or after the process. We considered aspiration pneumonia as a new cough, fever, and purulent sputum after the procedure and changes in imaging methods.

Statistical analysis

We performed statistical analysis using SPSS version 22.0 (IBM Corp., Armonk, NY). We expressed continuous variables as mean ± standard deviation and categorical variables as number (%). To identify independent factors for complications, we applied logistic regression analysis. We obtained odds ratios (ORs) and 95% confidence intervals (CIs) using univariate (crude) and multivariate (adjusted) models. We used statistically significant variables in the univariate model in the multivariate analysis. We used Kaplan-Meier curves to estimate mortality over the first 30 days and one year following PEG. We assessed differences between survival curves using the log-rank test. We considered a p-value of less than 0.05 to be statistically significant.

## Results

The demographic data for the 644 patients included in the study are presented in Table 1. The mean age of the patients was 65.12 ± 15.42 years, and the majority of the patients (60.6%) were female. In the vast majority of patients, the PEG indication was due to neurological causes (71.5%), among which most were strokes. The PEG indication was malignancy in 16.1% of patients (such as head and neck cancer or oesophageal cancer) and ‘other’ reasons (such as trauma or poor general condition after surgery for non-malignant diseases) in 12.3% of patients. Data on the indications of patients are shown in Table 1.

**Table 1:** Data on demographic, comorbidities, antibiotic use, and anticoagulant/antiaggregant use. BMI: body mass index; COPD: chronic obstructive pulmonary disease; CKF: chronic kidney failure; PEG: percutaneous endoscopic gastrostomy; LMWH: low molecular-weight heparin. *mean ± standard deviation.

	n (%)
Total number of patients	644
Age (years)^*^	65.12 ± 15.42
Gender	
Female	390 (60.6)
Male	254 (39.4)
BMI (kg/m^2^)^*^	23.09 ± 2.62
Indication	
Neurological disorders	461 (71.6)
Stroke	238 (37.0)
Dementia	107 (16.6)
Parkinson’s disease	69 (10.7)
Motor neuron disease	47 (7.3)
Malignancy	104 (16.1)
“Other” reasons	79 (12.3)
Comorbid diseases	
Hypertension	173 (26.9)
Diabetes mellitus	153 (23.8)
Coronary artery disease	35 (5.4)
COPD	10 (1.6)
CKF	7 (1.1)
Antibiotic therapy	
Therapeutic use for prevalent infection	314 (48.8)
Prophylactic use for PEG placement	282 (43.8)
No antibiotics	48 (7.5)
Anticoagulant/antiaggregant therapy	
LMWH	271 (42.1)
Aspirin	165 (25.6)
Clopidogrel	73 (11.3)
Aspirin and LMWH	11 (1.7)
Aspirin and Clopidogrel	6 (0.9)
No therapy	118 (18.3)

Among the patients’ comorbidities, hypertension was most frequent (26.9%), followed by diabetes mellitus (23.8%). In addition, 314 (48.8%) patients used therapeutic antibiotics for an existing infection, 282 (43.8%) patients used them for prophylactic purposes, and 48 (7.5%) patients did not receive any antibiotic treatment (therapeutic or prophylactic) before the PEG procedure. The majority (81.7%) received anticoagulant/antiaggregant therapy, while 18.3% did not receive this treatment. Data on patients’ comorbidities and antibiotic and anticoagulant/antiaggregant use are provided in Table 1. In addition, propofol was used in 96.3% of patients for sedation before the procedure, while midazolam was used in 3.7%.

Adverse events

The overall complication rate in our study was 11.2%. The most common was a peristomal infection in 37 (5.7%) patients. In the vast majority of patients, the infection was controlled by medical treatment. A subcutaneous abscess developed in one patient, and thus the tube was removed, and PEG was re-administered after the infection was under control. In addition, 16 (2.5%) patients experienced tube dislodgement, while 9 (1.4%) patients had bleeding. In two of these patients, bleeding was controlled by endoscopic haemostasis (epinephrine injection), while in others it was controlled by conservative methods without the need for any interventional procedures.

Aspiration pneumonia occurred in 5 (0.8%) patients and regressed after medical treatment. Peristomal leakage happened in 7 (1.1%) patients, from one of whom the tube was removed and then reapplied. A new tube was placed in 51 (7.9%) patients whose tube was displaced for different reasons (including peristomal injection, tube dislodgement, peristomal leakage, and obstruction). Data on complications related to the PEG procedure are shown in Table 2.

**Table 2:** Complications and mortality.

	n (%)	
Complication		
Peristomal infection	37 (5.7)	
Tube dislodgement	16 (2.5)	
Bleeding	9 (1.4)	
Peristomal leakage	7 (1.1)	
Aspiration pneumonia	5 (0.8)	
Total complication	72 (11.2)	
Mortality	30 days	1 year
Neurological reasons	53 (8.2)	172 (37.3)
Malignancy	6 (0.9)	28 (26.9)
“Other” reasons	4 (0.6)	20 (25.3)
Total mortality	63 (9.7)	220 (36.4)

Our univariate risk-factor analysis for complications included age, gender, body mass index (BMI), PEG indications, antibiotic use status, anticoagulant/antiaggregant therapy status, and comorbidity parameters. In our multivariate analysis for peristomal infection, independent risk factors for peristomal infection were (a) not taking antibiotic treatment before the procedure (OR 13.683; 95% CI 6.274-29.839) and (b) diabetes mellitus (OR 2.704; 95% CI 1.284-5.695) (Table 3). Furthermore, dementia markedly increased the risk of tube dislodgement (OR 5.740; 95% CI 2.045-16.112) (Table 4). On the other hand, in our multivariate analysis, independent risk factors for bleeding complications were (a) the use of clopidogrel (OR 15.041; 95% CI 2.690-84.108); (b) simultaneous use of aspirin and clopidogrel (OR 79.174; 95% CI 4.871-1287.012); and (c) hypertension (OR 7.888; 95% CI 1.703-36.533) (Table 5).

**Table 3:** Univariate and multivariate logistic regression analysis of the predictors of peristomal infection. OR: odds ratio; CI: confidence interval.

Predictor	Univariate analysis		Multivariate analysis	
	OR (95% CI)	p-Value	OR (95% CI)	p-Value
Stroke	1.485 (0.762–2.894)	0.246	1.107 (0.516–2.376)	0.794
“Other” reasons	0.393 (0.093–1.668)	0.206	0.743 (0.156–3.534)	0.709
No antibiotic therapy	18.221 (8.678–38.260)	<0.001	13.683 (6.274–29.839)	<0.001
Diabetes mellitus	4.193 (2.136–8.231)	<0.001	2.704 (1.284–5.695)	0.009

**Table 4:** Univariate and multivariate logistic regression analysis of the predictors of tube dislodgement. CKF: chronic kidney failure; COPD: chronic obstructive pulmonary disease; OR: odds ratio; CI: confidence interval.

Predictor	Univariate analysis		Multivariate analysis	
	OR (95% CI)	p-Value	OR (95% CI)	p-Value
Dementia	5.343 (1.959–14.572)	0.001	5.740 (2.045–16.112)	0.001
Coronary artery disease	1.576 (0.562–11.806)	0.223	1.083 (0.611–15.544)	0.173
CKF	1.911 (0.783–61.020)	0.082	1.561 (0.657–65.557)	0.109
COPD	1.585 (0.546–38.527)	0.161	1.123 (0.448–37.956)	0.211

**Table 5:** Univariate and multivariate logistic regression analysis of the predictors of bleeding. LMWH: low molecular-weight heparin; OR: odds ratio; CI: confidence interval.

Predictor	Univariate analysis		Multivariate analysis	
	OR (95% CI)	p-Value	OR (95% CI)	p-Value
LMWH	0.169 (0.021–1.359)	0.095	0.458 (0.041–5.126)	0.526
Clopidogrel	10.423 (2.733–39.751)	0.001	15.041 (2.690–84.108)	0.002
Aspirin + Clopidogrel	15.750 (1.648–150.548)	0.017	79.174 (4.871–1287.012)	0.002
Hypertension	3.475 (0.922–13.093)	0.066	7.888 (1.703–36.533)	0.008

Mortality

**Figure 2:**
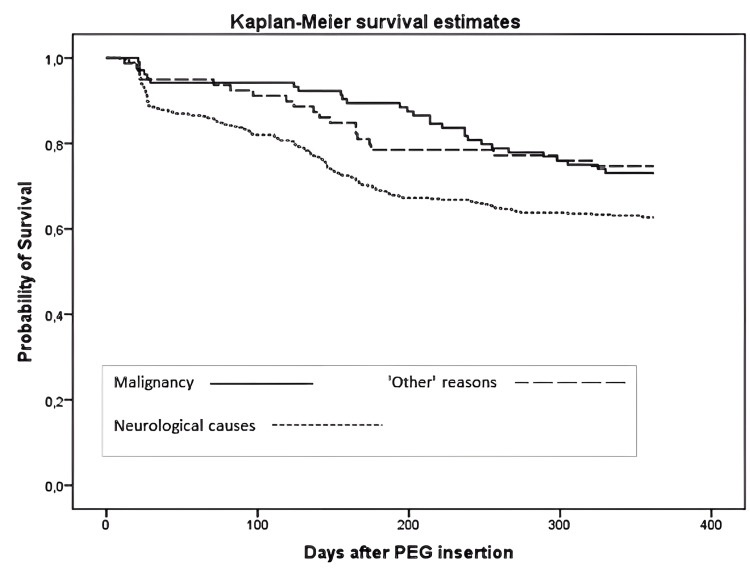
One year survival curve after PEG insertion stratified into three main causes for PEG, that is, malignancy, neurological disease and 'other reasons'. PEG: percutaneous endoscopic gastrostomy.

In our study, after the PEG procedure, the 30-day mortality rate was 9.7%, and the one-year mortality rate was 36.4% (Table 2). Fortunately, no patient died due to the PEG procedure. In addition, when the causes of death were classified as neurological, malignancy, or ‘other’, there was no significant difference between the groups in terms of 30-day mortality risk (p > 0.05). By contrast, the one-year mortality risk was statistically significantly higher in patients who underwent PEG due to neurological causes compared to those who underwent PEG due to malignancy or other reasons (p = 0.021 and p = 0.038, respectively) (Figure 2).

## Discussion

In this study, neurological diseases (71.6%) were the most common indication for the PEG procedure, followed by malignant diseases (16.1%). However, there were differences in previous studies with regard to the PEG indication. In the study conducted by Vujasinovic et al., the PEG indications were neurological diseases in 52% of the patients and malignant diseases in 32% [14]. Schneider et al. found that the rate of neurological disorders among the PEG indications was 29.4%, and the rate of malignant diseases was 54.6% [15]. We think that this difference may be due to the effect of hospital conditions, patient population, and the location of the hospital. For example, the absence of an oncology clinic in our hospital for a long period during our study may explain the low rate of malignant diseases in our study.

In our study, the overall complication rate was 11.2%. There are significant differences among the studies in the literature, both in terms of follow-up and complication definitions. For example, in a multicentre prospective cohort study, the follow-up period was 30 days, and the overall complication rate was 3.6% [16]. Udd et al. reported a follow-up period of one year and an overall complication rate of 23% [17]. Despite the differences between definitions, the PEG complication rates may be considerable. For example, in the study by Blomberg et al., the overall complication rate in the first two weeks after the PEG procedure was very high: 39% [10]. Therefore, determining the risk factors for PEG complications and being careful in patients with these risk factors are of great importance.

In our study, similar to others in the literature, the most common complication was peristomal infection (37 patients, 5.7%) [9]. The infection could not be controlled with medical treatment in only one patient; he or she developed a subcutaneous abscess. The patient’s PEG tube was removed, the abscess was drained, the infection regressed after appropriate medical treatment, and finally, the PEG procedure was repeated. In all other patients, the peristomal infection was controlled by medical treatment. In a prospective study conducted by Schneider et al., no patient had a peristomal infection, while Clarke et al. reported a very high rate of 38% [15,18]. The results of many studies in the literature are generally distributed between these two percentages [19,20].

In addition, in our study, not using antibiotics before the procedure and the presence of diabetes mellitus were independent risk factors for peristomal infection after the PEG procedure. Diabetes mellitus is considered an important risk factor associated with invasive procedures and postoperative wound infections. Indeed, it negatively affects immunity by suppressing polymorphonuclear leukocyte function and the cutaneous response to antigens [21]. Given that PEG is also an invasive procedure, the result of our study is not surprising.

We want to emphasize that more care should be taken with wound care for patients with diabetes mellitus. Moreover, discussions on antibiotic prophylaxis are still ongoing. While routine antibiotic prophylaxis is not recommended before a PEG procedure, according to ESPEN (European Society of Parenteral and Enteral Nutrition) guidelines, the results of a Cochrane systematic review supported the use of systemic antibiotics prior to the PEG procedure and showed that broad-spectrum antibiotics are effective against peristomal infection from the PEG procedure [13,22]. In addition, in a meta-analysis involving 10 randomized controlled trials, antibiotic prophylaxis applied before the procedure was reported to be effective in reducing the incidence of wound infection [23]. In our study, while the rate of peristomal infection was 2.9% in those using therapeutic antibiotics and 3.5% in those who received prophylactic antibiotics, this rate was 37.5% in those who did not use antibiotics. Therefore, we recommend using prophylactic antibiotics before the PEG procedure to protect against peristomal infection.

Inadvertent removal of a PEG tube is a common complication, with an incidence of about 4% in one study [9]. In a study involving 563 patients, its incidence increased to 12.8% [24]. In our study, the rate of tube dislodgement was 2.5%. We think that the reason for the relatively more reasonable tube-dislodgement rate in our study was that an experienced team performed the PEG procedure, and our country has highly performing home-health-care services. However, in our study, dementia was an independent risk factor for tube dislodgement. In fact, given some of the behaviours involved in dementia, this result was not surprising. Therefore, we would like to emphasize that the risk of tube dislodgement should be kept in mind for those with dementia.

In our study, the bleeding rate associated with the PEG procedure was 1.4%. While the rate of bleeding was 5.7% in the study by Pih et al., it was 1.0% in a multicentre prospective study involving 950 patients [16,25]. In contrast, in a prospective study by Schneider et al. [15], bleeding did not occur in any patient. This optimistic result can be attributed to the limited number of cases. In addition, the use of clopidogrel together with aspirin, as well as the use of clopidogrel alone, was an independent risk factor for bleeding complications associated with PEG in our study.

The American Society for Gastrointestinal Endoscopy (ASGE) evaluated the PEG procedure in the high-risk procedure class for bleeding and suggested that aspirin should be continued in people at risk for thromboembolism and that the use of clopidogrel should be stopped 7-10 days before the procedure [26]. Unfortunately, 22.7% of our patients using clopidogrel continued to use it on the day of the procedure. In fact, this finding may mean that guidelines have a limited effect on physicians’ preferences. A previous study reported that failure to comply with the guidelines might be a result of not being able to provide reminders during the operation rather than a negative opinion against those proposed in the guidelines [27].

One suggestion is to create a checklist and use it before each procedure. However, contrary to the results of our study and the suggestion in the guidelines, in a retrospective study involving 990 patients who underwent PEG, clopidogrel, and aspirin were not risk factors for bleeding [28]. We believe that scientists should illuminate this issue with prospective and multicentre studies due to a lack of consensus. Furthermore, our data suggested that hypertension was also an independent risk factor for PEG-related bleeding complications. As we did not find any information that corresponds to this result in the literature, we have approached this issue cautiously and think that it should be supported with future studies.

In our study, the 30-day and one-year mortality rates were 9.7% and 36.4%, respectively. Although some studies have shown mortality rates associated with PEG of up to 2%, none of the deaths in our study were associated with the PEG procedure or its complications [19,25,29,30]. We think this outcome was due to the experience of the physicians who performed the procedure and the successful patient care after the procedure. Considering that the 30-day mortality rate in the literature is between 4.8% and 20%, the result in our study can be considered reasonable [15-17,20]. Furthermore, in our study, there was no significant difference between the indications (in terms of neurological causes, malignancy, and ‘other’ causes) with regard to 30-day mortality risk.

Contrary to this result, Vujasinovic et al. reported that the risk of 30-day mortality was higher in those whose PEG indication was ‘other’ causes [14]. In addition, the one-year mortality rate in our study was in line with some studies in the literature [17,18]. However, in addition to optimistic results such as 17.4%, results up to 50% have been reported [19,20]. We think this difference may be due to dissimilarities in patient populations. Additionally, our study revealed that the one-year mortality risk in those who underwent PEG for neurological reasons was significantly higher compared to those who underwent the procedure for malignant disease and ‘other’ reasons. This result is in line with some studies in the literature [14,15,20]. Unlike in a prospective study involving 535 patients, those who underwent PEG for neurological disease and ‘other’ reasons had a higher risk of mortality than those who underwent PEG for malignant diseases [10].

The present study had some limitations. The first was that the hospital where we conducted our study is a university hospital. Patients in a university hospital are generally those with more comorbidities (such as diabetes mellitus, stroke, coronary artery disease, chronic kidney failure, chronic obstructive pulmonary disease, etc.) and more severe conditions. This factor may have an impact on some results. Another limitation of our study was that it was designed retrospectively. Thus, some patient information may have been recorded incorrectly or incompletely, a phenomenon that may result in inaccessibility for all patients and a bias risk. Nevertheless, the high number of patients and the large amount of data collected about patients and procedures increase the power of our study. In addition to our contribution to the literature with this large case series study, we think we have provided some useful information to clinicians who perform the PEG procedure, such as complications associated with the procedure, risk factors for complications, and mortality status.

## Conclusions

The PEG procedure is a safe and feasible technique due to its low complication and mortality rate in patients with swallowing disorders who need long-term nutritional support. Prophylactic antibiotics should be used to prevent peristomal infections. Furthermore, concomitant use of aspirin and clopidogrel, or just clopidogrel use, is a risk factor for bleeding. On the other hand, the data in our study showed that diabetes mellitus is a risk factor for peristomal infection, and hypertension is a risk factor for bleeding. In dementia patients, tube dislodgement should be kept in mind during patient care due to its high risk.

## References

[1] Gauderer MW, Ponsky JL, Izant RJ Jr (1980). Gastrostomy without laparotomy: a percutaneous endoscopic technique. J Pediatr Surg.

[2] Rahnemai-Azar AA, Rahnemaiazar AA, Naghshizadian R, Kurtz A, Farkas DT (2014). Percutaneous endoscopic gastrostomy: indications, technique, complications and management. World J Gastroenterol.

[3] Sacks BA, Vine HS, Palestrant AM, Ellison HP, Shropshire D, Lowe R (1983). A nonoperative technique for establishment of a gastrostomy in the dog. Invest Radiol.

[4] Russell TR, Brotman M, Norris F (1984). Percutaneous gastrostomy: a new simplified and cost-effective technique. Am J Surg.

[5] Ljungdahl M, Sundbom M (2006). Complication rate lower after percutaneous endoscopic gastrostomy than after surgical gastrostomy: a prospective, randomized trial. Surg Endosc.

[6] Yuan Y, Zhao Y, Xie T, Hu Y (2016). Percutaneous endoscopic gastrostomy versus percutaneous radiological gastrostomy for swallowing disturbances. Cochrane Database Syst Rev.

[7] Silas AM, Pearce LF, Lestina LS (2005). Percutaneous radiologic gastrostomy versus percutaneous endoscopic gastrostomy: a comparison of indications, complications and outcomes in 370 patients. Eur J Radiol.

[8] Gomes CA Jr, Andriolo RB, Bennett C, Lustosa SA, Matos D, Waisberg DR, Waisberg J (2015). Percutaneous endoscopic gastrostomy versus nasogastric tube feeding for adults with swallowing disturbances. Cochrane Database Syst Rev.

[9] Hucl T, Spicak J (2016). Complications of percutaneous endoscopic gastrostomy. Best Pract Res Clin Gastroenterol.

[10] Blomberg J, Lagergren J, Martin L, Mattsson F, Lagergren P (2012). Complications after percutaneous endoscopic gastrostomy in a prospective study. Scand J Gastroenterol.

[11] Chong VH, Vu C (2006). Percutaneous endoscopic gastrostomy outcomes: can patient profiles predict mortality and weaning?. Singapore Med J.

[12] Wirth R, Voss C, Smoliner C, Sieber CC, Bauer JM, Volkert D (2012). Complications and mortality after percutaneous endoscopic gastrostomy in geriatrics: a prospective multicenter observational trial. J Am Med Dir Assoc.

[13] Löser C, Aschl G, Hébuterne X (2005). ESPEN guidelines on artificial enteral nutrition--percutaneous endoscopic gastrostomy (PEG). Clin Nutr.

[14] Vujasinovic M, Ingre C, Baldaque Silva F, Frederiksen F, Yu J, Elbe P (2019). Complications and outcome of percutaneous endoscopic gastrostomy in a high-volume centre. Scand J Gastroenterol.

[15] Schneider AS, Schettler A, Markowski A (2014). Complication and mortality rate after percutaneous endoscopic gastrostomy are low and indication-dependent. Scand J Gastroenterol.

[16] Anderloni A, Di Leo M, Barzaghi F (2019). Complications and early mortality in percutaneous endoscopic gastrostomy placement in lombardy: a multicenter prospective cohort study. Dig Liver Dis.

[17] Udd M, Lindström O, Mustonen H, Bäck L, Halttunen J, Kylänpää L (2015). Assessment of indications for percutaneous endoscopic gastrostomy--development of a predictive model. Scand J Gastroenterol.

[18] Clarke E, Pitts N, Latchford A, Lewis S (2017). A large prospective audit of morbidity and mortality associated with feeding gastrostomies in the community. Clin Nutr.

[19] Peveling-Oberhag J, Osman I, Walter D (2019). Risk factors for early and late procedure-related adverse events in percutaneous endoscopic gastrostomy: A single center, retrospective study. J Gastroenterol Hepatol.

[20] Agudo Tabuenca A, Altemir Trallero J, Gimeno Orna JA, Ocón Bretón MJ (2019). Mortality risk factors after percutaneous gastrostomy: who is a good candidate?. Clin Nutr.

[21] Joshi N, Caputo GM, Weitekamp MR, Karchmer AW (1999). Infections in patients with diabetes mellitus. N Engl J Med.

[22] Lipp A, Lusardi G (2013). Systemic antimicrobial prophylaxis for percutaneous endoscopic gastrostomy. Cochrane Database Syst Rev.

[23] Jafri NS, Mahid SS, Minor KS, Idstein SR, Hornung CA, Galandiuk S (2007). Meta-analysis: antibiotic prophylaxis to prevent peristomal infection following percutaneous endoscopic gastrostomy. Aliment Pharmacol Ther.

[24] Rosenberger LH, Newhook T, Schirmer B, Sawyer RG (2011). Late accidental dislodgement of a percutaneous endoscopic gastrostomy tube: an underestimated burden on patients and the health care system. Surg Endosc.

[25] Pih GY, Na HK, Ahn JY (2018). Risk factors for complications and mortality of percutaneous endoscopic gastrostomy insertion. BMC Gastroenterol.

[26] Anderson MA, Ben-Menachem T, Gan SI (2009). Management of antithrombotic agents for endoscopic procedures. Gastrointest Endosc.

[27] (2006). Enhancing the use of clinical guidelines: a social norms perspective. J Am Coll Surg.

[28] Richter JA, Patrie JT, Richter RP (2011). Bleeding after percutaneous endoscopic gastrostomy is linked to serotonin reuptake inhibitors, not aspirin or clopidogrel. Gastrointest Endosc.

[29] Sheehan JJ, Hill AD, Fanning NP, Healy C, McDermott EW, O'Donoghue DP, O'Higgins NJ (2003). Percutaneous endoscopic gastrostomy: 5 years of clinical experience on 238 patients. Ir Med J.

[30] Erdil A, Saka M, Ates Y (2005). Enteral nutrition via percutaneous endoscopic gastrostomy and nutritional status of patients: five-year prospective study. J Gastroenterol Hepatol.

